# Open-label pilot clinical trial of citicoline for fragile X-associated tremor/ataxia syndrome (FXTAS)

**DOI:** 10.1371/journal.pone.0225191

**Published:** 2020-02-13

**Authors:** Deborah A. Hall, Erin E. Robertson, Maureen Leehey, Andrew McAsey, Bichun Ouyang, Elizabeth Berry-Kravis, Joan A. O’Keefe

**Affiliations:** 1 Department of Neurological Sciences, Rush University, Chicago, IL, United States of America; 2 Department of Communication Sciences and Disorders, Northwestern, Chicago, IL, United States of America; 3 University of Colorado Denver, Aurora, CO, United States of America; 4 Department of Cell and Molecular Medicine, Rush University, Chicago, IL United States of America; 5 Department of Biochemistry, Rush University, Chicago, IL, United States of America; 6 Department of Pediatrics, Rush University, Chicago, IL, United States of America; Cardiff University, UNITED KINGDOM

## Abstract

Fragile X-associated tremor/ataxia syndrome (FXTAS) is a late onset neurodegenerative disorder that is characterized by tremor, cerebellar ataxia, frequent falls, cognitive decline, and progressive loss of motor function. There are currently no approved treatments for this disorder. The purpose of this study was to determine if citicoline was safe for the treatment of tremor and balance abnormalities and to stabilize cognitive decline in patients with FXTAS. Ten participants with diagnosed FXTAS were administered 1000 mg of citicoline once daily for 12 months. Outcome measures and neurological examination were performed at baseline, 3 months, 6 months, and 12 months. The primary outcome was the FXTAS Rating Scale score. Secondary outcomes included change in a battery of neuropsychological tests, an instrumented Timed up and go test, computerized dynamic posturography, 9-hole pegboard test, and balance confidence and psychiatric symptom questionnaires. Safety was also evaluated. Citicoline treatment resulted in minimal adverse events in all but one subject over the course of the study. There was a significant improvement in the Beck Anxiety Inventory (*p* = 0.03) and the Stroop Color-Word test (*p* = 0.03), with all other measures remaining stable over the course of 12 months. This open-label pilot trial of citicoline for individuals with FXTAS showed that it is safe and well tolerated in this population.

**Registration**: This trial was registered at ClinicalTrials.gov. Identifier: NCT0219710.

## Introduction

Fragile X-associated tremor/ ataxia syndrome (FXTAS) is characterized by tremor, cerebellar ataxia, frequent falls, cognitive decline, and progressive loss of motor function [1]. It is a late onset neurodegenerative disorder that occurs in carriers of a premutation expansion (55–199 CGG) in the *fragile X mental retardation 1* (*FMR1*) gene located on the X chromosome [1]. There are approximately 785,000 *FMR1* premutation carrier women and 314,000 men in the US and roughly 75% of men and 15% of women carriers will develop FXTAS in their lifetime [2–5]. There are currently no targeted therapeutic treatments for FXTAS. However, there are promising pharmaceutical approaches with potential symptomatic benefits based on their effectiveness in treating disorders that have significant phenotypic overlap with FXTAS.

Citicoline is an endogenous nucleotide and intermediate in the biosynthesis of structural membrane phospholipids. A known phospholipase A_2_ inhibitor, it has been used to treat neurodegenerative disorders associated with head trauma, stroke, brain aging, cerebrovascular pathology and Alzheimer disease (AD) [6] and is available for over-the-counter use. In a FXTAS *Drosophila* model, cytidine 5’diphospho-choline (citicoline) demonstrated the ability to reduce neuronal toxicity caused by CGG repeat *FMR1* mRNA by decreasing locomotion deficits and lethality [7]. Citicoline has been shown to improve cognitive performance in patients with AD and mild dementia when given at 1000 mg per day [6]. Currently there are no FDA approved medications for treatment of the motor and cognitive dysfunction in FXTAS patients. The purpose of this study was to determine the safety of citicoline in FXTAS. The medication regimen in this protocol was similar to those studies conducted for AD and stroke [6].

## Methods

### Study design and participants

This was a single-center open-label phase II trial to test the impact and safety of citicoline on neurological signs in ten participants with FXTAS. The primary outcome measure was safety, with secondary outcomes focused on motor and cognitive function. One year was chosen as the endpoint as this was thought to be an adequate interval to capture adverse events (AEs). Participants were recruited through the FXTAS clinic at Rush University Medical Center (RUMC). All participants gave informed consent. Participants were included if they had a confirmed CGG repeat size between 55–200, had a diagnosis of possible, probable, or definite FXTAS per the Jacquemont et al diagnostic criteria [4], could follow directions for study activities, and were ambulatory. Serum creatine kinase, complete metabolic panel, complete blood count, liver function tests, renal function tests, platelets and EKG had to be within normal limits for participation. Exclusion criteria included inability to consent, presence of severe renal or hepatic disease, abnormal creatine kinase and/or platelet count in the past six months, pregnancy, allergy/sensitivity to the citicoline or its formulations, concurrent participation in another clinical study, active substance use or dependence or serious illness. The study protocol was approved by the Rush University Institutional Review Board after IND exemption was granted by the FDA. The study was registered at ClinicalTrials.gov Identifier: NCT0219710.

### Intervention

The study medication was manufactured by Jarrow Formulas and purchased from the online retailer ProVitaminas and shipped to RUMC. Pill bottles were provided to all participants with instructions to take one capsule of 1000mg daily for 12 months by the study coordinator. There was no stratification or randomization. Participants were asked to keep all other medications unchanged for the duration of the study. Neurological examination and outcome measures were performed at baseline and at the end of months three, six, and twelve. Adverse events were monitored throughout the study. Participants were seen in the FXTAS Clinic at RUMC at five in-person visits. Three telephone visits were conducted at three weeks, nine weeks and nine months to inquire about any AEs.

### Clinical and Laboratory Evaluations

#### FXTAS Rating Scale and Neurological exam

The FXTAS Rating Scale (FXTAS-RS), which was designed to measure the severity of motor signs in FXTAS patients [8], was administered to each participant. The scale has 44 items and a total score of 226. For this study, improvement was defined as a 20% improvement on the FXTAS-RS. Neuropathic signs were tested using the Total Neuropathy score modified to exclude nerve conduction velocity testing [9]. FXTAS stage was noted at baseline [10].

#### Neuropsychological tests

Participants were administered a battery of neuropsychological tests measuring several cognitive domains known to be affected in FXTAS, including: the Behavioral Dyscontrol Scale II (BDS-II) [11] and Stroop Color-Word test (Stroop CW) [12] for response inhibition; Wechsler Adult Intelligence Scale 3^rd^ Edition (WAIS-III) Full Intelligence Quotient (IQ) [13], Wechsler Adult Intelligence Scale Performance IQ (WASI PIQ) [13], Animal Naming [14] and the Controlled Oral Word Association Test (COWAT) [15] for verbal fluency; Digit Span for auditory attention and working memory [13] and the Symbol Digit Modalities test (SDMT) for information processing speed [16]. The oral version of the SDMT was used so that tremor or other deficits in fine motor coordination would not affect the test results. The Montreal Cognitive Assessment [17] was also administered as a measure of global cognitive function.

#### Motor testing

Participants performed a 7 meter instrumented Timed up and go (i-TUG; APDM^TM^; Oregon) gait test [18], computerized dynamic posturography (CDP) balance testing with the Sensory Organization Test (SOT) (Neurocom^TM^, Natus Medical Inc., 2009), and a 9-hole peg test for fine motor coordination [19].

#### Questionnaires

Participants were administered the Activities-specific balance confidence (ABC) scale [20], Beck Anxiety Inventory (BAI) [21], and Center for Epidemiological Studies Depression Scale Revised (CESD-R) [22].

#### Safety

Safety of the use of citicoline was determined based on participant reporting of AEs at study visits, neurological examinations, laboratory findings and EKGs.

### Data and statistical analysis

The primary outcome at one-year was the FXTAS Rating Scale score. Secondary outcomes included the ABC, BAI, i-TUG (specifically stride length and stride length variability scores), CDP Sensory Organization Test (SOT condition 5 where eyes are closed and proprioceptive input is unreliable), 9-hole pegboard test (right and left hands), and cognitive tests. Baseline and 12 months scores were done for efficacy using two-tailed tests with statistical significance declared at the 0.05 level. AEs were recorded and classified as to seriousness of the side effect and likelihood the side effect was related to the study medication. Frequency analysis was performed to determine treatment safety. Primary and secondary outcomes were analyzed using SAS (9.2). A linear mixed model with random intercept, random slope, and unstructured variance-covariance matrix was also performed for the primary outcome measure. Sample size calculations were not performed.

## Results

### Patients

**Fig 1:**
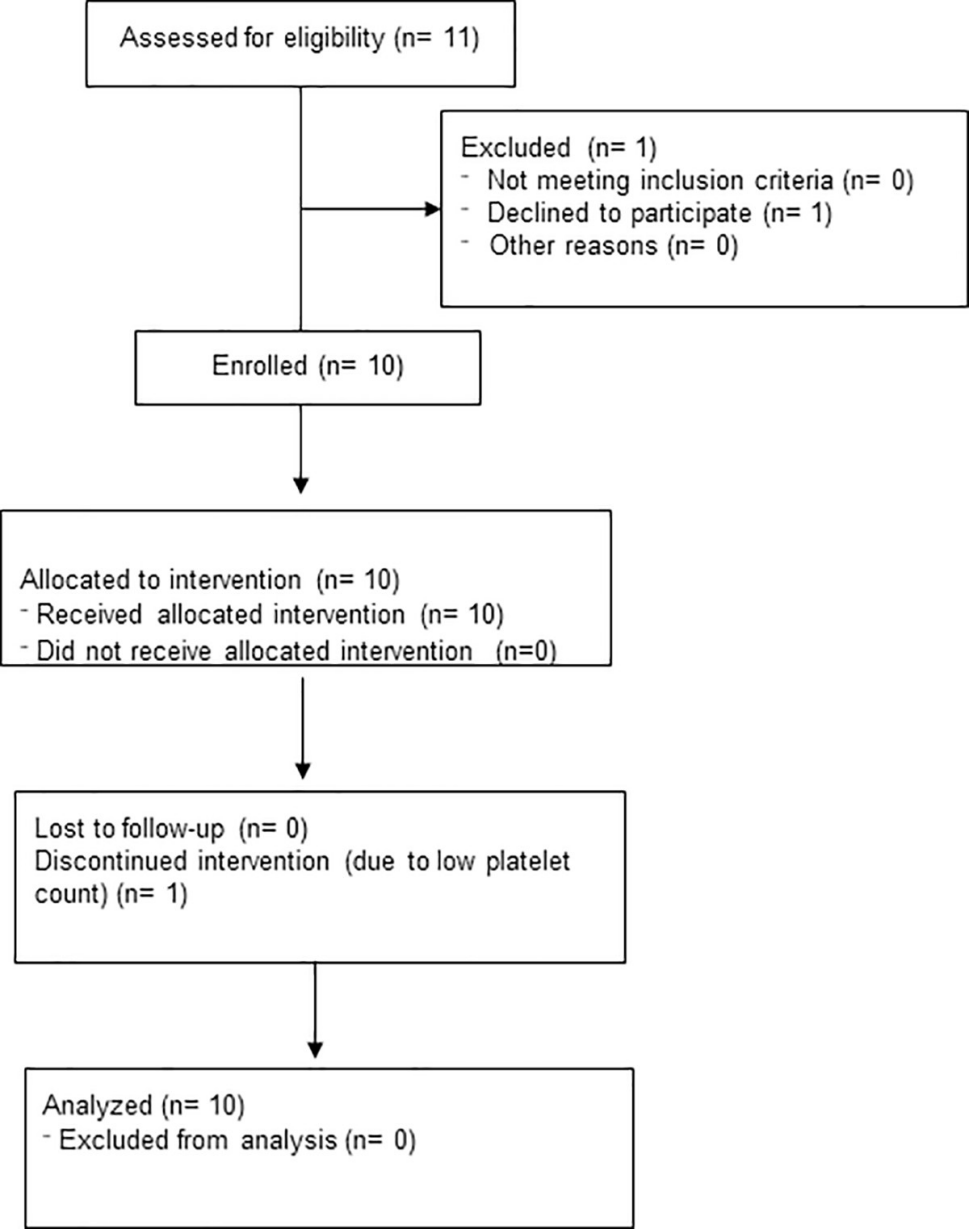
Consort flow diagram for recruitment.

All participants were recruited from 5/4/2015 to 12/17/2015 and seen for study visits 5/5/2017 to 1/20/2018 (**Fig 1**). Demographic information is presented in **Table 1**. All participants were white and non-Hispanic; there were nine men and one woman. This distribution is expected as there is a lower penetrance of FXTAS in women from X-inactivation. The subject with probable FXTAS was also the female subject. She was lacking neuro-radiographic findings consistent with FXTAS, but it is commonly seen in affected women that they do not have white matter abnormalities or moderate brain atrophy. Nine participants completed the one-year follow-up and one participant with a low platelet count withdrew prior to the end of the study and outcome data was missing for this participant at 12 months, but all available data were used for analysis.

**Table 1:** Baseline demographic and clinical characteristics.

Variable	N	Mean (SD)	%
**Age (years)**	10	70.00 (7.33)	
**Education (years)**	10	15.80 (2.53)	
**CGG repeats**	10	91.50 (15.57)	
**FXTAS-RS**	10	40.30 (21.16)	
Neuropathy scale*	10	0.50 (1)	
MoCA*	10	28.00 (5.00)	
**Sex**			
Women	1		10
Men	9		90
**FXTAS Diagnosis**			
Possible	0		10
Probable	1		0
Definite	9		90
**FXTAS Stage**			
1	3		30
2	2		20
3	1		10
4	3		30
5	1		10

*Median (interquartile range) is reported

### Efficacy

**Fig 2:**
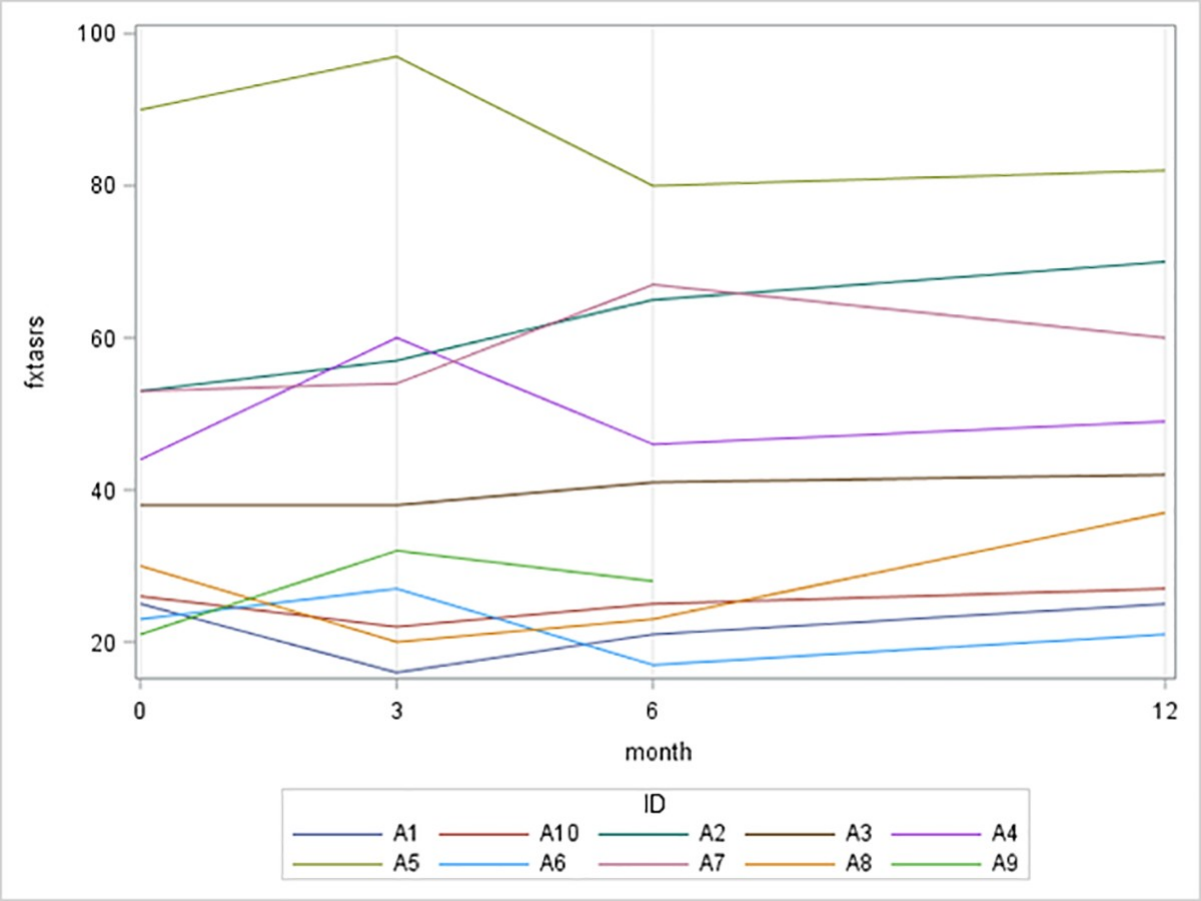
Plot of Fragile X-associated tremor/ataxia syndrome scores.

The primary outcome measure was the change in the total FXTAS Rating Scale score before and after treatment (**Table 2**, **Fig 2**). Secondary outcome measures included the ABC, BAI, CESD-R, stride length and its variability, SOT 5, 9-hole peg test, and cognitive tests. After 12 months, none of the participants demonstrated a 20% improvement on the FXTAS-RS, but scores were not significantly worse from baseline at the one-year follow up. Increasing scores of the FXTAS-RS represent worsening of the motor features of the disease. There was a significant decrease or improvement in BAI scores (*p* = 0.03) and significant increases or improvement in Stroop CW scores (*p* = 0.03). A linear mixed model with adjustment of age testing the difference between baseline and 12 months was fit and BAI remained significant (*p* = 0.02) while Stroop CW was not (*p* = 0.05). There were no other significant changes in any of the other secondary outcome measures.

**Table 2:** Secondary outcome measures at baseline and twelve months.

Outcome Measure	Baseline (n = 9)	12 Months (n = 9)	p-value
**FXTAS-RS**	** **	** **	** **
Mean (SD)	42, 44 (21.26)	45.8 (21.22)	0.18
Median (IQR)	38 (27)	42 (33)	
Min, Max	23, 90	21, 82	
**ABC**	** **	** **	** **
Mean (SD)	74.99 (16.57)	70.7 (20.64)	**0.39**
Median (IQR)	79.38 (13.75)	68.13 (26.8)	** **
Min, Max	46.25, 98.25	34.1, 96.9	** **
**BAI**	** **	** **	** **
Mean (SD)	9.33 (4.15)	3.89 (5.21)	**0.03**
Median (IQR)	9 (6)	3 (6)	** **
Min, Max	3, 16	0, 16	** **
**CESD-R**	** **	** **	** **
Mean (SD)	8.78 (6.34)	7.89 (9.57)	0.72
Median (IQR)	11 (11)	4 (4)	
Min, Max	1, 18	0, 30	
**BDS-II**	** **	** **	** **
Mean (SD)	23.22 (2.05)	22.11 (3.89)	0.28
Median (IQR)	24 (2)	23 (7)	
Min, Max	19, 26	17, 27	
**Stroop CW (n = 7)**	** **	** **	** **
Mean (SD)	26.86 (19.54)	35.14 (11.22)	0.03*
Median (IQR)	31 (16)	34 (14)	** **
Min, Max	15, 43	17, 52	** **
**WAIS-PIQ**	** **	** **	** **
Mean (SD)	106.89 (11.08)	107.44 (14.91)	0.8
Median (IQR)	105 (22)	107 (18)	
Min, Max	94, 121	86, 129	
**WAIS Full IQ**	** **	** **	** **
Mean (SD)	109.11 (11.11)	112.67 (14.68)	0.24*
Median (IQR)	107 (12)	112 (20)	
Min, Max	93, 127	93, 133	
**Digit Span (n = 8)**	** **	** **	** **
Mean (SD)	11.38 (4.1)	10 (3.12)	0.53*
Median (IQR)	9.5 (4)	9.5 (4)	
Min, Max	8, 20	7, 16	
**Animal Naming**	** **	** **	** **
Mean (SD)	94.78 (18.53)	95.33 (28.28)	0.92
Median (IQR)	98 (17)	77 (47)	
Min, Max	67, 131	67, 142	
**SDMT**	** **	** **	** **
Mean (SD)	77 (14.48)	79.11 (15.58)	0.41
Median (IQR)	79 (16)	81 (2)	
Min, Max	53, 99	55,97	
**COWAT**	** **	** **	** **
Mean (SD)	82.44 (16.84	79.78 (16.43)	0.4
Median (IQR)	80 (23)	82 (22)	
Min, Max	60, 110	5, 103	
**9 hole peg RH**	** **	** **	** **
Mean (SD)	39.41 (12.8)	41.67 (19.98)	1.00*
Median (IQR)	35.96 (5.2)	36.43 (7.33)	
Min, Max	22.08, 66.05	21.43, 91.75	
**9 hole peg LH**	** **	** **	** **
Mean (SD)	46.61 (30.72)	50.13 (43.04)	0.82*
Median (IQR)	35.49 (8.93)	37 (9.23)	
Min, Max	24, 27	25.78, 162.65	
**SOT5 (n = 5)**	** **	** **	** **
Mean (SD)	34.6 (31.66)	36.68 (33.83)	0.75*
Median (IQR)	55 (57)	56 (58.7)	
Min, Max	0, 61	0, 68.7	
**Stride length (%stature) mean**	** **	** **	** **
Mean (SD)	77.54 (5.38)	75.24 (9.3)	0.26
Median (IQR)	76.26 (5.34)	72.22 (7.85)	
Min, Max	71.92, 89.32	63.19, 96.06	
**Stride length (%stature) CoV**	** **	** **	** **
Mean (SD)	0.1 (0.03)	0.11 (0.02)	0.52
Median (IQR)	0.11 (0.04)	0.1 (0.05)	
Min, Max	0.06, 0.16	0.08, 0.14	

*p-value is based on Wilcoxon signed rank test.

**Key:** RH, right hand; LH, left hand; SOT5, Sensory Organization Test 5 score; CoV, Coefficient of Variation.

### Safety and tolerability

Safety was a secondary outcome and all participants were included in the safety analysis. Adverse events are presented in **Table 3** and are reported by frequency of participants reporting an adverse event. Eight participants experienced AEs over the course of the study. The participant that discontinued citocoline early had low platelets at six months. On record review, the participant had chronically low platelet levels prior to study entry and this was not felt to be related to study medication. One participant experienced diarrhea, which was thought possibly related to the study medication, as well as multiple falls and Meckel’s diverticulum, both of which were not attributed to the study medication. Another participant had an episode of acute vertigo, which was deemed possibly related to the study medication, and a third had esophageal varices that were determined to be unrelated to the medication. Three participants had falls, but these were not attributed to the study medication. One fall was associated with acute vertigo and the patient was diagnosed with benign positional vertigo and treated in the emergency department. One participant experienced temporary leg swelling and another had an increase in skin bruising, both of which were thought unlikely to be related to the study medication.

**Table 3:** Summary of adverse events and serious adverse events.

Adverse Events	Severity	N (%)*
**Type of Adverse Event**		
Diarrhea	Mild	2 (20.0)
Bruising	Mild	2 (20.0)
Vivid dreams	Mild	2 (20.0)
Dizziness	Mild	1 (10.0)
	Moderate- Severe	1 (10.0)
Vomiting	Moderate- Severe	2 (20.0)
Leg Swelling	Mild	1 (10.0)
Elevated Liver Enzymes	Mild	1 (10.0)
Weight Gain	Mild	1 (10.0)
**Serious Adverse Events**		
Gastrointestinal Disorders	Severe	2 (20.0)
Falls	Severe	3 (30.0)
Immunological Abnormality	Severe	1 (10.0)

*Frequency of participants with adverse event

## Discussion

This is the third clinical trial in individuals with FXTAS. Citicoline was selected due to its prior use in the treatment of other neurodegenerative disorders, its positive effect on cognition in AD [6], and the benefit seen in the FXTAS *Drosophila* model [7]. The primary aim of this study was to determine the effect of citocoline on motor symptoms. At one year, it was determined that citocoline did not result in a significant change in the FXTAS-RS, although worsening is generally expected in this population.

Only two of the secondary outcome measures changed significantly over 12 months. There was a significant decrease in BAI scores as well as significant increase in Stroop CW scores. It is possible that the decrease in anxiety was simply the result of participants becoming more comfortable at the study visits rather than an effect of the study medication. The improvement in Stroop CW scores suggests that the study medication may have improved response inhibition. A deficit in the Stroop CW test which tests inhibitory control has been shown in *FMR1* carrier men [23], although the impact of improvement in this cognitive measure alone is unclear. Another explanation for the improvement could be a learning effect. The lack of significant change in other secondary measures may suggest that the medication may have helped to stabilize disease progression overall. However, it must be noted that there were multiple comparisons in this study with unadjusted p-values due to the pilot nature of this study and the results, especially the statistically significant results, may simply be false positives.

It was also determined that citicoline was safe and well tolerated given that none of the serious adverse events could be clearly attributed to the study medication. This is also consistent with the over-the-counter availability of this compound in the United States. In addition, other secondary outcome measures mostly remained stable over the course of the year.

Citicoline was chosen because in the FXTAS *Drosophila* model it reduced the neuronal toxicity caused by expanding CGG repeat *FMR1* mRNA thereby decreasing locomotion deficits and lethality [7]. Phospholipase A_2_ inhibition by citicoline may similarly provide a mechanism to mitigate locomotion and cognitive deficits in FXTAS patients. Biomarkers have not yet been developed in FXTAS for use in clinical trials, but would be beneficial in future studies of this compound.

A caveat to the design of this study is the lack of a placebo arm. However, data from the first clinical trial in FXTAS investigating memantine suggests that the placebo effect in FXTAS is minimal, if present at all [24], and having a placebo group may not have altered the conclusions in this study. The FDA requested an open-label, small study due to a lack of long term data of the compound in similar populations. A second caveat is that the FXTAS-RS is under final validation and some of the items that were used in this study may not remain in the final version of the scale. This work is underway with our research team, but may also impact future studies. Furthermore, compliance with the pill counting was not included in this study and may be needed in this patient population in future clinical trials. This study did show that it is feasible to recruit and retain participants with this neurodegenerative disease for a one-year clinical trial and longer follow-up periods may be possible, if needed.

## Conclusion

This was the third clinical trial conducted in FXTAS. The results show that citicoline is safe to use in this population. Preliminary data suggests it may stabilize disease progression, but a larger study is warranted to confirm these results.

## Supporting information

S1 File Appendix A: Pilot trial of citocoline for FXTAS schedule of events. (DOCX)

S2 File Pilot trial of citocoline for FXTAS study protocol. (PDF)

S3 File TREND statement. (PDF)
