# Supplementary material for: Open-label pilot clinical trial of citicoline for fragile X-associated tremor/ataxia syndrome (FXTAS)
Source: PLoS One. 2020 Feb 13;15(2):e0225191. doi: 10.1371/journal.pone.0225191 (PMC7018079; doi:10.1371/journal.pone.0225191)
Supplement: S1 File — (DOCX) [file pone.0225191.s001.docx]

**Appendix A. Schedule of Events**

| **Evaluation** | **Screening** | **Baseline** | **Week 3** | **Week 6** | **Week 9** | **Month 3** | **Month 6** | **Month 9** | **Month 12** |
| --- | --- | --- | --- | --- | --- | --- | --- | --- | --- |
| Informed Consent | X |  |  |  |  |  |  |  |  |
| Documentation of FXTAS | X |  |  |  |  |  |  |  |  |
| Medical/Treatment Hx | X |  |  |  |  |  |  |  |  |
| Clinical Assessment |  | X |  | X |  | X | X |  | X |
| Neurological Exam |  | X |  | X |  | X | X |  | X |
| Adherence Assessments |  |  | X | X | X | X | X | X | X |
| Questionnaires |  | X |  |  |  | X | X |  | X |
| FXTAS-RS and other rating scales |  | X |  |  |  | X | X |  | X |
| Beck Anxiety/ CESDS-R scales |  | X |  |  |  | X | X |  | X |
| Mini Mental State Exam (MMSE)_ | X |  |  |  |  |  |  |  |  |
| Neuropsychological battery of tests |  | X |  |  |  | X | X |  | X |
| Computed Posturography/iTUG |  | X |  |  |  | X | X |  | X |
| EKG |  | X |  |  |  |  |  |  |  |
| Lab tests | X |  |  |  |  | X | X |  | X |
