# Supplementary material for: Open-label pilot clinical trial of citicoline for fragile X-associated tremor/ataxia syndrome (FXTAS)
Source: PLoS One. 2020 Feb 13;15(2):e0225191. doi: 10.1371/journal.pone.0225191 (PMC7018079; doi:10.1371/journal.pone.0225191)
Supplement: S2 File — (PDF) [file pone.0225191.s002.pdf]

PROTOCOL

Principal Investigator: **Deborah A. Hall, MD PhD**

Version date: **1/12/2015**

Study Title
Citicoline for neurological signs in fragile X-associated tremor/ataxia syndrome (FXTAS)

Objectives
The objective of this study is to determine if citicoline is effective for balance abnormalities and to stabilize cognitive decline in patients with FXTAS.

Design and Outcomes
The design of this study is an open label study conducted at one center. The primary outcome is change in the FXTAS Rating Scale (FXTAS-RS). Secondary outcome measures include neuropsychological tests (Behavioral Dyscontrol Scale, Stroop, WAIS PIQ, WASI, Animal Naming, Purdue Pegboard, Digits Backwards, Symbol Digits, and COWAT), instrumented Timed up and go (iTUG) test, Computed Dynamic Posturography (CDP), Activities-specific balance confidence (ABC) scale, Beck Anxiety Inventory (BAI), and Center for Epidemiological Studies Depression Scale Revised (CESD-R).

Interventions and Duration
The intervention will be 1000mg of citicoline once daily. Outcome measures and neurological examination will be performed at baseline, 3 months, 6 months, and 12 months.

Sample size and population
Sample size for the initial pilot study is ten patients. All patients will meet critieria for possible, probable, or definite FXTAS per the Jacquemont et al diagnostic criteria (Jacquemont, 2003). There will be no stratification or randomization.

STUDY OBJECTIVES

Primary Objective

The hypothesis tested is that citicoline will result in improvement of scores on the FXTAS Rating Scale after 12 months of treatment. The null hypothesis is that there will be no difference between baseline and final outcome measures on the FXTAS Rating Scale. This scale will be performed by the principal investigator, a movement disorder neurologist involved in the development of this scale.

Secondary Objectives

There are two secondary hypotheses. First, we hypothesize that subjects will have stabilization of cognitive decline on citicoline. This hypothesis will be tested by measuring scores on the following neuropsychological tests: Behavioral Dyscontrol Scale, Stroop, WAIS PIQ, WASI, Animal Naming, Purdue Pegboard, Digits Backwards, Symbol Digits, and COWAT. Second, we hypothesize that patients will improve on Computed Dynamic Posturography and the instrumented Timed up and go test. The secondary outcomes will be performed by the study team and led by the site investigator.

BACKGROUND

Rationale
Fragile X-associated tremor/ ataxia syndrome (FXTAS) is characterized by tremor, cerebellar ataxia, frequent falls, cognitive decline and progressive loss of motor function. It is a newly recognized, late onset neurodegenerative disorder that occurs in carriers of a premutation in the fragile X mental retardation 1 (*FMR1*) gene located on the X chromosome. There are approximately 785,000 female and 314,000 male *FMR1* premutation carriers in the US and roughly 75% of men and 15% of women carriers will develop FXTAS in their lifetime. There are currently no targeted therapeutic treatments for FXTAS. However, there are promising pharmaceutical treatment approaches with potential symptomatic benefits based on their effectiveness in treating disorders that have significant phenotypic overlap with FXTAS.

Citicoline is an endogenous nucleotide and intermediate in the biosynthesis of structural membrane phospholipids and has been shown in initial studies to be effective in treating neurodegenerative disorders associated with head trauma, stroke, brain aging, cerebrovascular pathology and Alzheimer disease (Alvarez, 1999). FXTAS occurs in some carriers of a premutation size expansion (55-200 CGG repeats) in the promoter region of the *FMR1* gene. Increased CGG repeat length leads to increased *FMR1* mRNA, which is implicated in the neuronal toxicity causing motor and cognitive dysfunction in FXTAS. In a FXTAS *Drosophila* model, citicoline demonstrated the ability to reduce neuronal toxicity caused by CGG repeat *FMR1* mRNA by decreasing locomotion deficits and lethality (Qurashi, 2012). Citicoline also improved cognitive performance in patients with Alzheimer disease and mild dementia when given at 1,000 mg per day (Alvarez, 1999). Currently there are no FDA approved medications for treatment of the motor and cognitive dysfunction in FXTAS patients

The study drug regimen in this protocol is similar to those studies conducted for Alzheimer disease and stroke. The active study drug will be dosed once daily due to convention. There are no aspects of the proposed dosing schedule that are not FDA-approved for other indications. This study is of high priority due to the absence of any proven medications to treat the symptoms of this disorder.

STUDY DESIGN

This is an open label designed study. Citicoline was chosen because of its novel mechanism of action and its preliminary success in treating some forms of neurological

disease. This will be the first clinical trial testing citicoline in patients with FXTAS. Citicoline has demonstrated the ability to mediate neuronal toxicity presumably via its inhibition of phospholipase A_2. This action of phospholipase A_2 inhibition may provide a mechanism to mitigate locomotion and cognitive deficits in this patient population.

SELECTION AND ENROLLMENT OF SUBJECTS

Inclusion criteria:

- Subjects will be diagnosed with Fragile X-associated tremor/ataxia syndrome (FXTAS) by the Jacquemont et al diagnostic criteria (see Table 1).
- Serum creatine kinase, complete metabolic panel, complete blood count, liver function tests, renal function tests, platelets and EKG are within normal limits (results obtained from primary care physician and dated within the past 6 months or obtained at screening visit).
- Older than age 18.

Exclusion criteria:

- Patient refuses to assent or no LAR if cognitively impaired
- Presence of severe renal disease (BUN 50% greater than normal or creatinine clearance <60 mL/min) or hepatic disease.
- Abnormal creatine kinase and/or platelet count in the past 6 months (as determined by lab reports obtained from primary care physicians or conducted at baseline).
- Women of childbearing potential who are pregnant at the time of screening or who will not use adequate protection during participation of the study.
- Allergy/sensitivity to the citicoline or its formulations.
- Concurrent participation in another clinical study.
- Active substance use or dependence.
- Serious illness (requiring systematic treatment/or hospitalization) until the subject either completes therapy or is clinically stable on therapy, in the opinion of the site investigator, for at least 60 days prior to study entry.

Table 1: Diagnostic criteria for FXTAS (adapted from Jacquemont et al, 2003)

Molecular	*FMR1* CGG Repeat Size 55-200
Clinical	
Major signs:	Intention tremor
	Gait ataxia
Minor signs:	Parkinsonism
	Moderate to severe short term memory deficits
	Executive function deficits
Radiological	
Major signs:	MRI white matter lesions in the middle cerebellar peduncle (MCP sign)

Minor signs:	MRI white matter lesions in cerebral white matter<br>Moderate to severe generalized atrophy
Diagnostic Categories	
**Definite:**	Presence of one major radiological sign plus one major clinical symptom
**Probable:**	Presence of either one major radiological sign plus one minor clinical symptom or<br>has two major clinical symptoms
**Possible:**	Presence of one minor radiological sign plus one major clinical symptom

Study Enrollment Procedures

Patients with Fragile X-associated tremor/ataxia syndrome (FXTAS) will be recruited through the Parkinson Disease and Movement Disorder clinic at Rush University Medical Center. There are nine movement disorder attending physicians and two fellows who see about 25 FXTAS patients a year as new patients and in follow up. Recruitment goals are expected to be predominantly achieved through the cohort of individuals seen at Rush, but advertising may also be conducted through monthly FXTAS meetings to enhance enrollment. Potential subjects may be identified by their treating physician in the Movement Disorders Clinic at Rush or referred to Rush from elsewhere. If a patient is interested in enrolling in the study, they will be invited to an initial study visit, at which they will sign informed consent after a full explanation of the study.

Documentation of inclusion and exclusion criteria will occur if the patient is interested in enrollment. Eligible subjects who refuse participation will be given the option of later enrollment if they should choose to join the study.

Much of recruitment will occur from physicians with a treatment relationship with the patient. Consent will occur between the primary investigator on the study and the potential subject. All investigators are trained in the conduct of human research. The patient will have a mini mental state examination (MMSE) performed to determine level of understanding prior to the consent process. If the potential subject scores less than 25, the patient will not be allowed to gain entry into the study. A copy of the consent form will be provided to all subjects.

STUDY INTERVENTIONS

Interventions, Administration, and Duration

Ten subjects will receive 1,000mg once daily of citicoline. Each subject will stay on the study drug for 12 months. The outcome measures will be assessed at baseline and then again at the end of month 3, month 6, and month 12. The subjects will continue taking the same study drug at the same dose throughout the entire trial. There are no expected side effects, however, adverse events will be monitored throughout the study. Subjects will be seen and treated in the outpatient Movement Disorders Clinic at Rush. Subjects will come to the clinic for 6 in person visits and 3 telephone visits will be conducted. All patients may remain on other medications for neurologic signs, but the dose of these medications needs to remain stable during the 12 months of the study.

The Handling of Study Interventions

The study drug will be manufactured by Jarrow Formulas and purchased from the online retailer ProVitaminas in the form of 250mg capsules. The study drug will then be shipped to the Principal Investigator at Rush. The study drug will be dispensed by the study team at Rush who will dispense the study drug to the subjects. The study drug will be stored at room temperature in the Parkinson Disease and Movement Disorder clinic which will be secured by lock. The study drug will be provided to the subjects in pill bottles. They will receive four capsules of 250mg daily for the 12 months of intervention. Subjects should take four capsules once a day at mealtime, or two capsules twice a day at mealtime if they are having upset stomach. Subjects will be asked to return any unused study drug after 12 months to Rush. Study intervention accountability records will be completed according to the Manual of Operations.

Concomitant Interventions
Medications for neurological signs of Fragile X-associated tremor/ataxia syndrome need to be stable during the 12 months of this study. There are no contraindicated interventions.

- Required Interventions: citicoline 1,000mg once daily.
- Prohibited Interventions: none.

Adherence Assessment
Adherence will be assessed by pill counts and adherence questionnaires that will be completed at each of the study visits. Because the study is using an intent to treat analysis, adherence will be reported using descriptive statistics.

CLINICAL AND LABORATORY EVALUATIONS

Timing of Evaluations
Subjects will be consented and have initial laboratory studies and rating scales at the baseline visit. Once on the study drug, they will be contacted by phone at Week 3 for questions regarding side effects, clinical issues, and to answer any questions. The subjects will be reassessed after six weeks for anxiety and depression, and they will continue with the intervention. Again, they will be contacted by phone at Week 9 and Month 9 to assess for safety and tolerability. They will be seen in the clinic at Month 3, Month 6 and Month 12and outcome measures will be performed.

Table 2

**Evaluation**	**Screening**	**Baseline**	**Week 3/TC**	**Week 6**	**Week 9/TC**	**Month 3**	**Month 6**	**Month 9/TC**	**Month 12**
	Visit 1	Visit 2	Visit 3 /TC	Visit 4	Visit 5 /TC	Visit 6	Visit 7	Visit 8 /TC	Visit 9
Informed Consent	X								
Documentation of FXTAS	X								
Medical/Treatment Hx	X								
Clinical Assessment		X		X		X	X		X
Neurological Exam		X		X		X	X		X

Adherence Assessments			X	X	X	X	X	X	X
Questionnaires		X				X	X		X
FXTAS-RS and other rating scales		X				X	X		X
Beck Anxiety/ CESDS-R scales		X				X	X		X
Mini Mental State Exam (MMSE)_	X								
Neuropsychological battery of tests		X				X	X		X
Computed Posturography/iTUG		X				X	X		X
EKG		X							
Lab tests	X					X	X		X
Safety and tolerability phone call					X			X	

Pre-Intervention Evaluations
These evaluations occur prior to the subject receiving any study interventions.

- Screening

At the screening visit, the subject will have diagnosis confirmed and medical history reviewed. Inclusion and exclusion criteria will be completed.

- Pre-Entry

Screening and pre-entry may occur concurrently. Consent will be obtained. EKG and blood tests will be completed at the visit.

On-Study Evaluations
Blood chemistries, liver function tests, and hematology (Comprehensive Metabolic Panel [CMP] and CBC with differential) will be performed at Month 3, Month 6 and Month 12. FXTAS Rating Scale, neuropsychological tests, Computed Dynamic Posturography, instrumented Timed up and go test, and rating scales for movement disorders will be performed four times during the study. The allowable time window is Schedule of Evaluations ±7 days.

- Baseline

The baseline (entry) visit will occur at least two weeks after the screening visit to allow for adequate study drug supply. Study drugs will be dispensed at the entry visit.

- Intervention Discontinuation Evaluations

Evaluations done at the time of discontinuation include blood chemistry, hematology, FXTAS-RS, neuropsychological tests, Computed Dynamic Posturography, instrumented Timed up and go test, and the rating scales. In addition, an adherence assessment will also be done.

- Post-Intervention Evaluations

Neurological examinations will be performed according to the standard of care by the treating neurologist/investigator.

- Final Evaluations

The final visit will consist of blood chemistry and hematology, neurological examination, FXTAS Rating Scale, neuropsychological tests, Computed Dynamic Posturography, instrumented Timed up and go test, rating scales, and adherence assessment.

Special Instruction and Definitions of Evaluations

The source document will include all of the following listed below.

- Informed Consent: The patient will have an opportunity to review the consent form and ask any questions prior to signing the form. If the patient has dementia, they will be excluded from the study. Documentation of the consent will be given to the subject, and copies will be maintained in the study chart and the hospital record.
- Documentation of FXTAS: Diagnosis of FXTAS will need to be documented prior to entry in the patient's chart.
- Medical History: The subject's history regarding FXTAS will be obtained or a record of this history secured for the study chart. A complete social history, family history, and review of systems will also be performed.
- Treatment History: Allergies will be reviewed. Prior and current medications and other treatments will be reviewed and documented.
- Clinical Assessments: Clinical assessment procedure will be reviewed at the baseline entry visit and neurological examination performed. Clinical assessments to review safety, tolerability, and effectiveness will be conducted at each face-to-face visit or over the phone. Neurological examination will occur at the face-to-face visits only. Change in neurological signs or symptoms, adverse events, or tolerability issues should be recorded on the CRFs.
- Laboratory Evaluations: Blood chemistry and hematology will be performed and the resulting laboratory result form placed in the study record.
- Questionnaires and Rating Scales: Scoring for each item on the FXTAS Rating Scale will be recorded in the scoring grid. Scoring for the neuropsychological tests, CDP, iTUG, ABC, BAI, CESDS-R, and MoCA will be recorded in the CRF.
- Adherence Assessments: Pill counts and questionnaires will be filled out at every phone or in person visit.

Off-Intervention Requirements

Recording of adverse events for subjects after the study will occur in the setting of regular care with the treating neurologist.

FXTAS Rating Scale

The FXTAS-RS was designed to measure the severity of motor signs of FXTAS, tremor, ataxia, and parkinsonism. The scale was developed from a combination of three separate measures: the Clinical Rating Scale for Tremor (CRST), the International Cooperative Ataxia Rating Scale (ICARS), and the Unified Parkinson's Disease Rating Scale (UPDRS). The resultant scale has 44 items and a total score of 226. The tremor sub-

domain (score range 0-53) assesses action and postural tremor, drawing, and handwriting. The ataxia sub-domain (0-73) assesses posture and gait, limb ataxia, dysarthria, and oculomotor disturbances. The parkinsonism sub-domain (0-100) assesses bradykinesia, gait and balance, rest tremor, and rigidity (Tassone, 2010). The FXTAS-RS is relatively safe and simple to administer. It uses a quantitative scale format that has strong internal consistency and good inter- and intrarater reliability with different patient populations, including geriatric patients. It has specifically been clinically validated for evaluating motor signs in FXTAS patients.
For this study, improvement will be defined as a 20% improvement on the FXTAS-RS.

MANAGEMENT OF ADVERSE EXPERIENCES
There are no common adverse events with citicoline in adult studies. Adverse events will be documented on the adverse event form and sent to the independent medical monitor. Due to its pharmacological effects on the dopaminergic system, citicoline may enhance the effects of L-Dopa. Therefore, any subjects taking L-Dopa during the study will be monitored very closely for adverse events (EFSA, 2013).

CRITERIA FOR INTERVENTION DISCONTINUATION
Criteria and rules for stopping subject treatment include: new toxicological findings or serious adverse events, the patient withdraws consent without need for justification, the patient is no longer able to participate for medical reasons (such as surgery), if eligibility criteria are being violated, the subject develops significant neuropsychiatric symptoms such as suicidal ideation or psychosis, or if the subject fails to comply with the protocol. Subjects who stop treatment will still be asked to complete a final visit with rating scales and blood work.

STATISTICAL CONSIDERATIONS

General Design Issues
The primary and secondary outcomes will be analyzed using SPSS. The independent medical monitor will review adverse event data and the final analysis. There will be no interim analysis performed. There will be no stratification.

Outcome and Data Analysis
- Primary outcome (Aim 1): The primary hypothesis will be tested using a paired t-test using means of the total FXTAS Rating Scale score before and after treatment. Linear regression will be used to determine if there is an association among outcome, gender, age, race, or ethnicity. This outcome will be determined by the rating of patients from a blinded movement disorders neurologist.
- Secondary outcomes (Aim 2): Secondary hypotheses will be tested using paired t-tests on the neuropsychological tests, CDP, iTUG, and the ABC scale.
- Side effects (Aim 3): Adverse events will be recorded and classified as to seriousness of the side effect and likelihood the side effect is related to the study drug. For side effect analysis, frequency analysis will be done.
  Sample Size and Accrual: Sample size was estimated using published means and standard deviations of the FXTAS-RS in FXTAS subjects. To allow for attrition, 10

subjects will be recruited. Intent to treat analysis will be performed. The number of subjects lost to follow-up is likely to be low due to the relative lack of efficacy of other forms of treatment for this particular symptom of FXTAS. For the FXTAS-RS outcome, a sample size of 58 patients is needed for a 20% effect size, using a mean score of 36.6 and a standard deviation of 11 with a mean change of -7.3, based on a power of 80% and α=0.5. However, the first phase of testing is a pilot study looking at study methods and safety. We will not have adequate sample size to determine efficacy, but will be able to use the pilot data to plan the larger phase III study.

Data Monitoring

An independent medical monitor will be identified to monitor this study. An interim safety analysis will not be performed. Guidelines for stopping the study would include new toxicological findings of citicoline or serious adverse events reported from the subjects on active medication. The independent medical monitor will meet with the PI as needed during the protocol.

DATA COLLECTION, SITE MONITORING, AND ADVERSE EXPERIENCE REPORTING

Records to Be Kept

All study forms will be kept at Rush University. The records will be stored in a locked office of the site investigator. At the main site, data on the CRF will be entered into a password protected database. Access to the database will be restricted to the host site CRA, PI, and biostatistician.

Quality Assurance

The PI will meet with the study coordinator once monthly to review records, consent forms, protocol compliance, and data quality. All of the investigators will meet once every four months to review issues related to quality.

Adverse Experience Reporting

Adverse events will be recorded on the adverse event CRF and reported within three days. Serious adverse events will be reported within 24 hours. Detailed definitions of adverse experiences, a table for grading their severity, and details of how clinical sites are to report them appear in the Manual of Operations.

HUMAN SUBJECTS

Institutional Review Board (IRB) Review and Informed Consent

This protocol and the informed consent documentation and any subsequent modifications will be reviewed and approved by the IRB responsible for oversight of the study. A signed consent form will be obtained from the subject. Subjects who cannot consent for themselves cannot be included in the study. The consent form will describe the purpose of the study, the procedures to be followed, and the risks and benefits of participation. A copy of the consent form will be given to the subject, or legal guardian and this fact will be documented in the subject's record.

Subject Confidentiality
All laboratory specimens, evaluation forms, reports, video recordings, and other records that leave the site will be identified only by the Study Identification Number (SID) to maintain subject confidentiality. All records will be kept in a locked file cabinet. All computer entry and networking programs will be done using the SIDs only. Clinical information will not be released without written permission of the subject, except as necessary for monitoring by IRB, the FDA, the NINDS, the OHRP, the sponsor, or the sponsor's designee.

Study Modification/Discontinuation
The study may be modified or discontinued at any time by the IRB, the NINDS, the sponsor, the OHRP, the FDA, or other government agencies as part of their duties to ensure that research subjects are protected.

PUBLICATION OF RESEARCH FINDINGS

Publication of the results of this trial will be governed by the policies and procedures developed by the Principal Investigator.

FUTURE PLANS

The proposed study constitutes a phase II clinical trial. Upon completion of the study our plan is to conduct a phase III clinical trial of citicoline.

REFERENCES CITED

1. Alvarez XA, Mouzo R, Pichel V, Perez P, Laredo M, Fernandez-Novoa L, Corzo L, Zas R, Alcaraz M, Secades JJ, Lozano R, Cacabelos R. Double-blind placebo-controlled study with citicoline in APOE genotyped Alzheimer's disease patients. Effects on cognitive performance, brain bioelectrical activity and cerebral perfusion. Methods and Findings in Experimental and Clinical Pharmacology. 1999; 21(9): 633-644.
2. Qurashi A, Liu H, Ray L, Nelson DL, Duan R, Jin P. Chemical screen reveals small molecules suppressing fragile X premutation rCGG repeat-mediated neurodegeneration in Drosophila. Human Molecular Genetics. 2012; Vol 21, No. 9: 2068-2075.
